# The Innervation of the Tooth-Pulp

**Published:** 1899-02

**Authors:** G. Carl Huber

**Affiliations:** Ann Arbor, Mich.


### THE DENTAL REGISTER.

VOL. LIII.] FEBRUARY, 1899. [No. 2.

### COMMUNICATIONS.

### The Innervation of the Tooth-Pulp.

BY G. CARL HUBER, M.D., ANN ARBOR, MICH.

Read before the Tri-State Dental Meeting, at Put-in-Bay, Ohio, June, 1898.

In glancing through the literature of the microscopic anatomy of the tooth, one is early impressed with the want of unanimity in the views expressed by various writers.

Owing to the difficulty which surrounds investigations on development of tooth structures, many interpretations have been made on these questions. Indeed, it is well known that, in order to obtain good sections of tooth pulp *in situ*, one must be conversant with histological methods, both difficult and tedious. It is not my purpose, however, to burden you with an account of the microscopic anatomy of the tooth as a whole, but to bring to your notice some observations on one of the most debatable points in tooth histology, namely, the mode of ending of the nerves which enter the tooth-pulp.

This question is one fraught with many difficulties, and the reason it has been so variously answered by dentists and scientists is the want of proper staining methods. Our knowledge of the nerve-endings, both sensory and motor, has in recent years been greatly advanced by the more general introduction of two staining methods, the Golgi chrome-silver method and the Ehrlich methylen-blue method.

The value of these methods is found in the fact that in successfully stained preparations, the nerve fibres to their finest terminations, are differently stained, and may be therefore distinguished from the other tissues; from this fact much new light has been thrown on their finer structure and their relation to other elements.

It has long been the hope of a number of investigators that these methods might be successfully employed in the study of the nerves of the tooth-pulp. Such attempts have, as a general rule, given only negative results. Recently, however, Retzius has been able to stain the pulp nerves after the Golgi method, and my observations are based on successfully stained methylen-blue preparations. Before giving these observations, I deem it profitable to review briefly the results obtained by other writers on this subject.

The first authentic reference to an observation on the mode of ending of the pulp nerves which I have been able to find is that given by Boll in 1868. He worked on the tooth-pulps of rabbits and guinea-pigs. He made out numerous medullated and non-medullated nerve fibres in the tooth-pulp, with an especially dense net-work of non-medullated fibres under the odontoblasts, from this he traced fine fibrillae between the odontoblasts, but could never follow such fibrils into the dentinal tubules, but he regarded such a distribution as certain, even though the proof was lacking. He assumes that in the dentin there are found two kinds of tubules, such as contain the dentinal fibres and such as contain the nerve fibres.

Recent papers by Morgenstern describes bundles of axis-cylinders, surrounded by thin, medullary sheaths, which run into the dentin to certain, well-defined places. The nerves are said to run through tubules of different sizes which can be distinguished from the ordinary dentinal tubules by the ordinary methods of research. Each canal is said to contain at least two axis-cylinders which terminate in. the dentin in knob-shaped structures, and in the enamel in the following ways: (a) The axis-cylinder may pass through the entire nerve corpuscle, to terminate in its periphery. (b) The axis-cylinder may terminate on a nucleus of the nerve corpuscle. (c) The axis-cylinder may pass through the entire nerve corpuscle, winding itself around one or several nuclei, to end on the last nucleus of the so-called corpuscle. These nerve corpuscles are most fully developed in the bicuspids and in the molars, in the former particularly in the cusps, the latter by the side of the cusps.

Rose, in experiments, did not agree with Morgenstern, he claimed Morgenstern's end corpuscles are only areas of uncalcified ground substances, through which pass the dentinal fibres.

Tomes very correctly states that "it seems unlikely that there are in the dentin special canals, some larger and some smaller than the dentinal tubules, that these should not have been noticed. The dentinal tubules being remarkable for their uniformity of diameter, then, too, these end corpuscles must have occupied spaces which would be conspicuous in dry dentin sections." That medullated nerve fibres should pass beyond the odontoblasts into the dentin seems to me very improbable, as such nerves might be easily detected in ordinary tested preparations, certainly in those stained in osmic acid. It seems quite clear that Morgenstern must have been in error.

Mummery, also, by following Boll's method of preparation, by Weil's process, and also by decalcification processes, has convinced himself that a great number of fibres pass from the pulp to the dentin, these fibres being much smaller than the dentin fibres properly so-called, therefore confirming Boll's researches. He has not, however, succeeded in seeing what becomes of them when they reach the dentin.

Many other observers, White, Coleman, Hopewell Smith, Legros, Magitot, and Bodecker are also in line with those quoted above, differing to some extent in stating methods and on a few minor points, but they all believe in a continuity between the odontoblasts and the nerves of the pulp; they voice the opinions more generally held at the present time.

In answer to these observations, certain general considerations may not be out of place. In the first place, it must be borne in mind that the odontoblasts are differentiated, connective tissue cells of mesoblastic origin. It is assumed by the above writers that these connective tissue cells perceive, originate, or at least transmit impressions or stimuli, which become sensations when transferred to the pulp-nerves. Assuming that the nerves of the pulp are connected with the odontoblasts, we would have here a physiological phenomenon without parallel in nerve physiology. Secondly, I recall no instance where it is clearly shown that a sensory nerve fibre becomes continuous with any kind of cell, either its body nucleus or any of its branches, excepting, of course, the cell body of the neuron, of which said sensory fibre is a part. But all observations of recent times show that the end fibres of sensory nerves terminate between the cellular elements of the tissue in which they end. These considerations, it seems to me, warrant the theoretical conclusion that the pulp-nerves are not directly connected with the odontoblasts, or any other of the pulp-cells. I shall endeavor in the remaining portion of this paper to show that observations made with the special nerve stains, Golgi method and methylen-blue method, support this theoretical conclusion.

Retzius, in three late papers, has published observations a little in advance of what had been known before. In the first paper he deals with the ending of the pulp-nerves in fishes and reptiles. In the fish the pulp-nerves terminate in fine fibrillae under the dentin, no nerve fibres traced into the dentinal tubules. In the second paper he deals with the ending of pulp-nerves in amphibia. Here also the pulp-nerves terminate in fine, varicose fibrillae in the neighborhood of the dentin, often immediately under it, never in the dentin. In the third paper he treats of the ending of the pulp-nerves in the mammalia. Successful preparations were obtained with the Golgi method from the teeth of young mice. No nerve fibres were traced into the dentin but fine fibrilli were traced to the odontoblast zone, and between the odontoblasts. So we have a freed ending of the pulp-nerves between the odontoblasts, and between these cells and the dentine.

In my own investigations the pulps of the lower cuspids and molars of dogs, cats and rabbits were used, and in all I was able to stain the nerves. After some time, however, it was found that the pulp-nerves were more readily sained in the rabbit, consequently my observations were confined to the lower molars of these animals.

By cutting away the thin layer of dentin from the anterior and posterior surfaces of one of the molars the pulp may be readily removed by inserting a fine needle under one of the processes of the pulp and making some slight traction. The pulps removed in this way were found hardly crushed or lacerated, and with the greater portion of odontoblast layer in place.

The staining method employed in this investigation is, as already stated, the *intra-vitam* methylen-blue method. In the present research its application was as follows: A dog, cat or rabbit was killed with chloroform, and as soon as death ensued the carotid on one side was exposed and a canula inserted. Through the canula there was injected enough of a 1 percent solution of methylen-blue, made up in normal salt solution, to cause the tongue and lips to assume a deep blue color. About thirty minutes after the lower jaw was removed and teeth removed by aid of bone forceps, care being taken not to injure the pulp. The pulps were removed as above described and placed on a slide moistened with normal salt solution. Such preparations after standing awhile the axis-cylinders of the nerves are stained deeply blue, the other tissues not at all or faintly blue. As these preparations fade quite rapidly I use as a fixative a saturated solution of ammonium picrate, or a solution of ammonium molybdate. The former mounted in glycerol, the latter hydrated and mounted in balsam.

In all nearly one hundred well stained pulps were studied, of these the ones taken from the molars of rabbits were the best. They will, I think, apply equally well to the pulp-nerves of man. It is hardly necessary to state that the methylen-blue method, as above described, cannot be employed in the study of pulp-nerves of the human teeth.

The nerve fibres going to the pulp are, as is well known, medullated nerve fibres. They approach the lower portion of the pulp in one or several large nerve bundles. On reaching the lower surface of the pulps these larger nerve bundles break up into numerous smaller ones, these consisting on the average of eight or ten nerve fibres, although larger and smaller ones are met with. These form a plexus of medullated fibres in the fibrous tissue membrane which covers the under surface of the pulp. Not all nerve fibres terminating in the pulp are found in the small bundles of medullated fibres above referred to. Many medullated fibres leave the nerve bundles and approach the surface of the pulp near the lower edge of the odontoblast layer. Many fibres as they approach the lower edge of the odontoblast layer, lose their medullary sheath and break up into numerous non-medullated branches. These non-medullated branches, as I have been repeatedly able to observe, pass up into the pulp immediately under the layer of odontoblasts, having a very regular, almost straight course. That they are non-medullated fibres is readily made out by their varicosity. Their ultimate ending will be considered at another place.

Your attention is again drawn to the medullated fibres which ascend in the substance of the pulp. In preparations of the whole pulp, cleared so as to be quite transparent, it may be observed that medullated fibres, or small bundles consisting of two or three such fibres, from place to place become separated from larger bundles found in the substance of the pulp and wend their way toward its surface. On approaching the surface of the pulp the medullated fibres lose their medullary sheath; the non-medullated terminal branches, after repeated division, form a plexus immediately under the odontoblasts. This plexus is not the dense plexus described by many observers, and is a plexus only in so far as that the varicose nerve-fibrils cross each other in various directions, forming thus an interlacing net-work. I am inclined to think that observers who have described a dense plexus of nerve fibres under the odontoblasts must have mistaken connective tissue fibres and processes of pulp cells of the odontoblasts for nerve fibres. I find no evidence, for instance, for considering Weil's layer as a layer of non-medullated fibres, as suggested by Hopewell Smith.

As to the ultimate ending of the pulp-nerves, the point most disputed, my own observations corroborate most fully those made by Retzius with the Golgi method. I find, as he did, that terminal fibrils given off from the plexus of a varicose fibre found under the odontoblasts pass up between these cells, to terminate usually in fine granules near the free end of the odontoblasts.

Now and then some small fibril may be traced as it takes a tangential course over the freed ends of the odontoblasts, as Retzius also found.

All my observations, taken in connection with those given by Retzius, warrant, it seems to me, the statement that the terminal branches of the pulp-nerves end between the odontoblasts near their free surface, occasionally between these cells and the dentin. They do not, I believe, make any connection with the odontoblasts nor with any of the cellular elements of the pulp. In all preparations examined by me I was unable to trace any nerve fibril beyond the odontoblast layer, as one might expect if the nerve fibres extended into the dentin, in which case we might expect, I think, that on removing the pulp here and there some nerve fibril would be pulled out of the dentin and might be observed extending beyond the odontoblast layer. In conclusion, then, it might be stated, by way of summary, that methylen-blue preparations show that the sensory fibres entering the tooth-pulp end in rather simple end-brushes consisting of long, varicose fibrillae, which are found under the odontoblasts, terminating in free endings, and are in connection with the dentinal fibres directly or through the odontoblasts.

If, then, the nerve fibres do not enter the dentin or are not continuous with the dentinal fibres, how is it we experience pain when the dentin is operated upon?

Black says that it required the pulp and peridental membrane to make up the sum of the sensory functions of the tooth. I have thus far made no reference to nerves of the peridental membrane. Retzius, in one of his papers, refers to such nerves. From theoretical considerations, I should say the nerves of the peridental membrane were stimulated mechanically; that is, by pressure. The peridental membrane may thus be regarded as the organ of touch of the tooth. I don't mean by this that there are special tactile corpuscles, I find no evidence for this. The pulp-nerves cannot be stimulated by pressure.

Dr. Black states the pulp-nerves are temperature nerves only, in the sense that they are stimulated by changes in temperature, the resulting sensation being one always of pain. Whether in the excavation of a cavity enough heat is developed to act as a stimulus to the pulp-nerves, is purely a matter of speculation. I can imagine that such may be the case when operating on a sensitive tooth, especially when the bur is used. I am well aware that numerous objections may be made to such a theory.

That, as a result of the laceration and injury which they must undergo when the dentin is operated upon, some metabolic changes may not be set up in the odontoblasts, which, as I may venture to suggest, may have as the result the liberation of heat or possibly some chemical product which may stimulate the pulp-nerves terminating between them, I am not prepared to deny.

But this is not a conduction of impulses from the dentin to the pulp-nerves in the sense expressed by Coleman, Legros, Magitot, Smith, Robertson and Bodecker.

The odontoblasts are not end organs, as some of these investigators would have us believe, but differentiated mesoblastic cells, which have for their primary function the formation of dentine.

In this account stress has been laid on the anatomical findings, and I believe I may say that recent investigations with the Golgi and methylen-blue methods have thrown some light on that portion of the microscopic anatomy of the tooth which deals with the pulp-nerves. This portion of my paper I trust future investigation will uphold. As to the manner in which the pulp-nerves are stimulated we can at present only theorize, and I hope that further observations may soon lead to a better understanding of this portion of the question under consideration.

